# Aprepitant in a Multimodal Approach for Prevention of Postoperative Nausea and Vomiting in High-Risk Patients: Is There Such a Thing as “Too Many Modalities”?

**DOI:** 10.1100/tsw.2009.34

**Published:** 2009-04-28

**Authors:** John J. Hache, Manuel C. Vallejo, Jonathan H. Waters, Brian A. Williams

**Affiliations:** University of Pittsburgh, Department of Anesthesiology, Liliane S. Kaufmann Building, Pittsburgh, PA 15213, United States

**Keywords:** postoperative nausea and vomiting, PONV, prophylaxis, multimodal prophylaxis, aprepitant, 5-HT3 antagonists, perphenazine, emesis

## Abstract

Postoperative and postdischarge nausea and vomiting (PONV and PDNV, respectively) add morbidity to perioperative outcomes. Combining some antiemetic agents of different mechanisms is more effective than using single agents, although this concept has not yet been tested extensively with aprepitant. Consecutive high-risk patients for PONV (n = 100) were given preoperative aprepitant 40 mg before surgery and were followed perioperatively. Female patients receiving general anesthesia (n = 81) were selected for data analysis. The primary endpoints were PONV/PDNV in the 48 h after surgery. For patients included in the data analysis, using Apfel PONV risk factors, the median risk count was four out of four. PONV and PDNV incidences were 21% (95% CI: 14-31%) and 37% (95% CI: 27-48%), respectively. Two patients experienced PACU (postanesthesia care unit) vomiting and two patients experienced emesis postdischarge. When using regression modeling and comparing patients who received one or two vs. three or four mechanistically unique antiemetics (added to preoperative aprepitant), while adjusting for surgical case duration, the three or four additional antiemetic group showed more PONV/PDNV (Odds Ratio 3.73, 95% CI 1.3-10.9, *p* = 0.016) than did the one or two additional drug group. There were no other predictors of PONV/PDNV (transabdominal surgery, four vs. three Apfel risk factors) in these patients. The low incidence of vomiting (2-5%) suggests the potential importance of aprepitant in a multimodal antiemetic regimen. However, there may be the potential that too many unique antiemetic mechanisms combined with preoperative aprepitant may actually increase the incidence of perioperative nausea.

## INTRODUCTION

Postoperative nausea and vomiting (PONV) continues to present significant challenges to patient care and patient satisfaction. Patients fear PONV more than postoperative pain or gagging on the endotracheal tube[1,2]. Costs of PONV are estimated to be several hundred million dollars (U.S.$) annually, and these costs involve added morbidity resulting from aspiration, wound dehiscence, and unanticipated hospital admission. Furthermore, pneumomediastinum, pneumothoraces, and subcutaneous emphysema with airway compromise have also been reported[3,4,5,6]. Emphasizing the significance of PONV, a recent study reported that patients would be willing to pay $90 for effective PONV prophylaxis[7].

Combination therapy with multiple antiemetic medications has been shown to be more effective than a single agent alone, although the risk for PONV has yet to be totally eliminated[8]. Recently, an additional patented antiemetic, aprepitant, has become available. Its use is largely reserved for patients with multiple PONV risk factors, and its effectiveness and optimal use in high-risk patients has not yet been fully defined.

In this journal, our institution previously published a "zero-tolerance" approach for PONV prophylaxis based on a literature review of antiemetic agents[9]. Aprepitant was described only briefly in that review since it was just recently approved; since that review publication, select patients have been receiving aprepitant at our institution. As an initial attempt to determine the value of aprepitant use for our institution's patients, the present case series was initiated.

Aprepitant, a neurokinin-1 receptor antagonist, has been shown to be superior to ondansetron for prevention of vomiting in the first 24 and 48 h postoperatively[10]. However, superior efficacy of aprepitant for the prevention of nausea has not been demonstrated and is thought to be comparable to other antinausea options[11].

We evaluated PONV and postdischarge nausea and vomiting (PDNV) in 100 consecutive patients who were prescribed aprepitant preoperatively after evaluation in the preoperative clinic. The aim was to describe the incidence of PONV/PDNV up to 48 h after surgery when aprepitant was used in combination with other antiemetic medications in patients presenting for elective surgery.

## METHODS

This study was designed as an observational case series of patients at high risk (baseline risk of 60–80%) for PONV. Each patient was specifically categorized based on the number of PONV risk factors published by Apfel et al., including a history of PONV, female sex, nonsmoking status, and perioperative opioid use[14]. There were no additional interventions beyond routine care, since aprepitant is being prescribed to surgical patients with a history of PONV in the preoperative clinic at our institution, as part of the standard of care. Following IRB approval, 100 consecutive patients who presented to the anesthesia preoperative clinic were given a prescription for aprepitant, 40 mg, with instructions to take orally 3 h prior to surgery. These patients were followed perioperatively and categorized with respect to the aforementioned Apfel risk factors. Consensus guidelines for the prevention of PONV recommend that patients with three to four risk factors for PONV receive combination therapy with two to three mechanistically different antiemetic agents[12]. Out of the 100 patients completing the series follow-up, criteria for inclusion into the data analysis included preoperative aprepitant administration, female gender, a history of PONV, three or more Apfel risk factors for PONV, and the administration of at least one additional antiemetic agent other than aprepitant. Patients administered anesthesia other than general anesthesia, having received aprepitant as the sole antiemetic agent, male patients, and/or those receiving aprepitant without a history of PONV were excluded from data analysis after data collection.

The primary objective of the study was to describe the incidence of PONV in patients receiving aprepitant along with other antiemetic agents as part of a multimodal approach to the prevention of PONV. Secondary objectives included obtaining patient-reported evaluations of their PONV/PDNV outcomes.

Antiemetic drugs used in this study are routinely used at Magee-Women's Hospital for the management of patients undergoing anesthesia for surgery and treatment of PONV, as part of a standardized regimen for postoperative care at our hospital. Patients were evaluated for nausea using a visual analog scale (VAS); 0 = no nausea and 10 = worst nausea ever, and for the presence of emesis. For the purposes of data collection, patients reporting any degree of nausea were considered positive for PONV. After the establishment of PONV, the PONV policy of our institution is to initiate treatment with second-line agents, including promethazine, perphenazine, and metoclopramide. Other drugs on the formulary may be used as well based on the severity of symptoms and clinical judgment of the anesthesiologist. Perioperative analgesics, specifically opioids, were titrated to patient comfort based on the postoperative standardized protocol to have the VAS pain score (0 = no pain and 10 = worst pain ever) less than 4 on patient discharge. PACU (postanesthesia care unit) discharge was based on the Aldrete score[13].

All antiemetic doses, in addition to the type and duration of anesthesia, were recorded for each patient. Information regarding PDNV was obtained from a postoperative follow-up phone call to assess postoperative pain, nausea, and satisfaction with their procedure. In addition, there was a follow-up phone interview 1–2 weeks after surgery by a research assistant blinded to the intraoperative proceedings. The patients were asked: (1) how they were feeling (open-ended), (2) if there was any PDNV during the first 48 h, (3) if there was any persisting pain, (4) if they were satisfied, and (5) if they would likely request aprepitant in the future.

## STATISTICAL ANALYSIS

Descriptive statistics were used to describe demographic data, PONV risk factors, surgical data (length and type of surgery), use and type of perioperative antiemetics, and combined overall incidence of PONV/PDNV. PONV/PDNV data are presented with 95% confidence intervals (95% CI).

To address the question of whether "too many" unique antiemetic agents (combined with aprepitant) changed the rate of nausea, we used patient cases meeting the inclusion and exclusion criteria. We then explored cross-tabular data (using the Chi square test) involving the occurrence of any PONV/PDNV (up to 48 h) based on clusters of total antiemetic mechanisms used for prophylaxis. Logistic regression was used to determine the relationship between PONV/PDNV and the number of additional antiemetic agents (one or two vs. three or four, in addition to aprepitant), the number of Apfel risk factors (three vs. four), the type of surgery (transabdominal open procedure vs. all others), and length of time in the operating room (as a continuous variable).

Data analysis was conducted using GraphPad Prism 5 (GraphPad Software Inc., La Jolla, CA) and SPSS version 16.0 (SPSS, Inc., Chicago, IL).

## RESULTS

From the 100 patients evaluated, 81 met the criteria for inclusion into the data analysis. All included patients had a history of PONV. The average age was 49.4 years and 71 (of the included 81) patients had four Apfel[1,2] risk factors, while the other ten patients had three Apfel risk factors. The expected PONV rate would be expected to approach 80% in untreated individuals[14]. A present history of cigarette smoking (known to be protective against PONV in contexts of general anesthesia) was found in only 11% of the patients included in the data analysis.

Surgical procedures (Table 1) were varied. Laparoscopic surgery and weight loss surgery were common, as were gynecologic and breast surgery. Nineteen procedures were transabdominal and open (i.e., not laparoscopic), while the other 62 were not. The length of time in the operating room was an average of 189 min. All patients included in the data analysis received general anesthesia with volatile agents. Opioid use was typical, with 99% receiving fentanyl and 27% receiving morphine.

**TABLE 1**
**Anesthetic and Surgical Data**

| | |
|---|---|
| Type of surgery | n |
| Lap gastric bypass | 25 |
| Lap gastric banding | 13 |
| TAH/BSO | 12 |
| Breast surgery | 11 |
| Sacrocolpopexy/ colporrhaphy | 10 |
| Other procedures | 6 |
| Laparoscopy | 4 |
| Length of surgery (min ± SD) | 189 ± 112 min |

Abbreviations: n, number; min, minutes; lap: laparoscopic; TAH/BSO: total abdominal hysterectomy/bilateral salpingo-oophorectomy.

All 81 patients received aprepitant, with the majority (n = 57) receiving two or more antiemetic agents (Table 2). This resulted in 89% receiving ondansetron, 70% dexamethasone, 33% transdermal scopolamine, and 4% receiving granisetron or droperidol (Table 3). Overall, 61 patients received aprepitant and one to two additional mechanistically unique antiemetic drugs for prophylaxis, and the remaining 20 received three to four antiemetic agents (in addition to aprepitant). Only one patient received a fourth mechanistically unique agent (droperidol). The remainder of the patients in the group receiving three or more agents in addition to aprepitant received the combination aprepitant, ondansetron, dexamethasone, and transdermal scopolamine.

PONV and PDNV incidences (up to 48 h) are listed in Table 4. PONV was experienced in the PACU by 21% (95% CI: 14–31%) of patients and 2.5% (95% CI: 0.7–9%) experienced emesis. When accounting for recurring symptoms within individual patients, 37% (95% CI: 27–48%) experienced nausea and 5% (95% CI: 2–12%) experienced emesis, either in the PACU or during the first 48 h.

Table 5 shows the number of patients with PONV and PDNV based on number of risk factors (per Apfel's criteria). Of the patients with four risk factors, 39% (95% CI: 29–51%) experienced PONV/PDNV. Of the ten patients with three PONV risk factors, 20% (95% CI: 6–51%) experienced PONV/PDNV.

Surprisingly, 12 of 20 patients (60%) that received aprepitant plus three or four other antiemetics encountered nausea within the first 48 h after surgery vs. only 30% (18/61, $p = 0.014$ by the Chi square test) of patients who received aprepitant plus one or two other antiemetics. The discovery of increased PONV in this group was not an outcome from a specific aim of the study, but rather an unexpected finding. The two groups did not differ significantly with respect to the average number of risk factors for PONV (data not shown, $p = 0.26$).

In the group receiving aprepitant plus one or two additional antiemetic agents, 51% underwent gastric bypass or gastric banding, 16% breast surgery, 13% hysterectomy, 8% sacrocolpopexy, and 12% laparoscopy, cystoscopy, or other procedures, and the average time in the operating room was 189 min. For the group receiving aprepitant plus three or four additional antiemetic agents, 35% had gastric bypass or gastric banding surgery, 35% hysterectomy, 15% sacrocolpopexy, 10% breast surgery, and 5% laparoscopy, with an average time in the operating room of 191 min. There were no discernable differences based on type of surgery or mean duration in the operating room. A logistic regression analysis did show a trend toward increased PONV with increasing time in the operating room ($p = 0.053$, see below).

**TABLE 2**
**Number of Antiemetics Received and PONV/PDNV Incidence**

| Agent Combination | n (Patients) | PONV/PDNV Incidence |
|---|---|---|
| Aprepitant with 5-$HT_3$ antagonist only | 18 | 5/18 (28%) |
| Aprepitant with scopolamine only | 2 | 0/6 (0%) |
| Aprepitant with dexamethasone only | 4 | |
| Aprepitant with one or two of the listed antiemetic agents | 61 | 18/61 (30%)* |
| Aprepitant with three or more of the listed antiemetic agents | 20 | 12/20 (60%)* |
| Overall | | 30/81 (37%) |

* $p$ = 0.014 by Chi square test.

**TABLE 3**
**Perioperative Antiemetics**

| | % |
|---|---|
| Aprepitant | 100 |
| Ondansetron | 89 |
| Dexamethasone | 70 |
| Scopolamine patch | 33 |
| Other* | 4 |

% reported out of 100 total patients.

* Other antiemetics included granisetron and droperidol.

**TABLE 4**
**Incidence of PONV**

| | | |
|---|---|---|
| PACU nausea | 16 patients | 20% (95% CI: 13–30%) |
| PACU vomiting | 2 patients | 2.5% (95% CI: 0.7–9%) |
| PACU PONV | 17 patients | 21% (95% CI: 14–31%) |
| Nausea up to 48 h | 30 patients | 37% (95% CI: 27–48%) |
| Vomiting up to 48 h | 4 patients | 5% (95% CI: 2–12%) |
| PONV/PDNV up to 48 h | 30 patients | 37% (95% CI: 27–48%) |

Since all patients received aprepitant, nausea outcomes after one or two additional drugs were compared with the outcomes of patients receiving three or four additional drugs. Logistic regression analysis, after adjustment for length of surgery ($p$ = 0.053) revealed that patients in this series receiving aprepitant plus three or four additional antiemetic agents were more likely to experience PONV than patients receiving aprepitant plus one or two additional agents ($p$ = 0.016, Table 6).

Forty-nine patients responded regarding their impression of the anesthetic and antiemetic regimen administered. Of patients leaving narrative comments, 78% were favorable, describing either complete satisfaction or decreased PONV symptoms relative to previous anesthetics. Of patients with negative comments, 16% were related to complaints of nausea or vomiting, and 6% were related to postoperative

**TABLE 5**
**Nausea and Vomiting Sorted by Risk Factors**

| Number of Risk Factors | Number of Patients | PACU Nausea | PACU Vomiting | PACU PONV | Nausea up to 48 h | Vomiting up to 48 h | PDNV up to 48 h |
|---|---|---|---|---|---|---|---|
| 4 | 71 | 16 (22%) | 1 (1.4%) | 16 (22%) | 28 (39%) | 3 (4.2%) | 28 (39%) |
| 3 | 10 | 0 | 1 (10%) | 1 (10%) | 2 (20%) | 1 (10%) | 2 (20%) |
| Total | 81 | 16 (20%) | 2 (2.5%) | 17 (21%) | 30 (37%) | 4 (4.9%) | 30 (37%) |

**TABLE 6**
**Predictors of PONV/PDNV Based on Multivariable Logistic Regression***

| Predictor of PONV | Odds Ratio | 95% CI | *p* Value |
|---|---|---|---|
| Aprepitant plus three or more antiemetic agents | 3.73 | 1.29–10.9 | 0.016 |
| Time in operating room | 1.004 | 1.00–1.01 | 0.053 |

* Regression model based on type of surgery, number of antiemetic agents, number of risk factors for PONV, transabdominal surgery, and length of time (min) in the operating room.

All tested covariates were dichotomous (dummy) variables except for operating room time (continuous variable, in minutes). Factors that were not predictive in this model were number of Apfel risk factors (three vs. four) and open transabdominal surgery (vs. other, data not shown).

pain. Of the 30 patients experiencing PONV and/or PDNV, 14 (47%) of these patients had positive comments.

## DISCUSSION

In this case series, we present our experience administering aprepitant to a series of patients with three or more risk factors for PONV/PDNV. All of the patients included for data analysis received volatile anesthetics, had a history of PONV, and were female. Apfel et al. determined that the risk for PONV is predicted by the number of factors, specifically female gender, prior history of motion sickness or PONV, nonsmoking, and the use of perioperative opioids. If zero, one, two, three, or four of these risk factors were identified, the rate of PONV is predicted to be 10, 21, 39, 61, and 79%, respectively, without PONV prophylaxis[14].

Notably, this case series consisted of a population at very high risk for PONV, with a median of four risk factors. Without prophylaxis, the risk of PONV would be expected to approach 79%. The patients analyzed in this series had a PONV incidence of 21% (95% CI: 14–31%) in the PACU and 37% (95% CI: 27–48%) throughout the first 48 h. Given the number of administered antiemetic agents (two to five), these rates do not appear to differ from the rates predicted by Apfel et al., although their study followed patients for 24 h, whereas ours followed patients for 48 h. In either case, we found anecdotally through this case series that aprepitant improved PONV outcomes in our institution's population vs. not using aprepitant.

We did not expect to find increasing nausea when three or more agents with unique antiemetic mechanisms were coadministered with aprepitant. Since this finding was not our specific aim, further research is needed via a randomized controlled trial. In our 12 patients with nausea after three or four antiemetics, nine (75%) received the combination of ondansetron-dexamethasone-transdermal scopolamine, one received the combination of ondansetron-dexamethasone-droperidol, one received granisetron-dexamethasone-transdermal scopolamine, and one received ondansetron-dexamethasone-transdermal scopolamine-droperidol.

The screening process for being considered positive for PONV/PDNV symptoms was deliberately conservative. When asking patients if they had any symptoms, if they responded with "mild nausea" or "significantly decreased nausea compared to previous experiences", these were considered "positive" for PONV/PDNV. It is also possible that any symptoms such as dizziness or light-headedness may have been interpreted by the patients as very mild nausea, although it could also have been an unrelated complaint.

The type and length of surgery are also thought to contribute to PONV. Length of surgery approached an average of 3 h in our series and each hour of anesthesia time is known to increase PONV risk[8]. Laparoscopic, bariatric, and breast surgery were common among our series participants. However, only hysterectomy was independently associated with an increased risk of PONV among the patient procedures studied by Apfel et al.[8].

This case series data justify a formal randomized controlled trial including aprepitant as part of a multimodal antiemetic plan, as previously suggested in this journal by Skledar et al. and Williams et al.[9,15]. Specifically, future research should pay particular attention to the possibility of adverse multimodal interactions (specifically aprepitant with $5HT_3$-antagonist *plus* dexamethasone *plus* transdermal scopolamine). We speculate that transdermal scopolamine may be problematic in this multimodal scheme, since aprepitant-dexamethasone-$5HT_3$ antagonist combinations appear to be generally accepted for use in high-risk chemotherapy-induced nausea-vomiting patients; again, further research is needed.

Limitations of this study include its design as an observational case series. Being an observational series, there was no control arm and the patients were subject to the care of an anesthesiologist not guided by any particular study protocol. The lack of a standardized protocol allowed the majority of patients to receive aprepitant plus one or two additional antiemetic agents with a small number of patients receiving three or more agents in addition to aprepitant. This disproportionate distribution increases the possibility of chance findings impacting our data. In either case, this work serves as a preliminary series justifying well-controlled randomized studies where aprepitant is used as part of a multimodal antiemetic regimen. Another limitation of this observational study is that no data were collected for data analysis regarding the degree or severity of nausea or vomiting, other than patient comment. However, validated methods exist where symptom severity can be quantified[16,17].

It is difficult to determine how the described antiemetic protocol compares with the world literature on PONV prophylaxis. This protocol made significant advances in symptom reduction compared with our institution's previous benchmarks. However, more structured study is required in which high-risk patients routinely receive a combination such as ondansetron, dexamethasone, and perhaps a nonsedating antidopaminergic such as perphenazine[15,18], and the dependent variable entails the use of preoperative aprepitant vs. placebo. After such study, we should be better able to determine the incremental value of aprepitant when compared with multimodal off-patent agents (or therapeutic equivalents) defined in consensus guidelines.

## CONCLUSION

Combination PONV prophylaxis with aprepitant demonstrates a trend toward decreased incidence of PONV in ambulatory patients at high risk for PONV except when three to four drug combinations (most commonly a $5HT_3$-antagonist-dexamethasone-scopolamine) were used, in which case there was more nausea. As a result, the question of "how much is too much" appears to apply with respect to the number of unique antiemetic mechanisms coadministered with aprepitant. An important finding in our study was the low number of patients that experienced vomiting (two in PACU and four overall). In either case, our patients' anecdotal comments suggest that aprepitant as part of a multimodal approach to the prevention of PONV is associated with an acceptable level of patient satisfaction.

It has been previously published that 5-$HT_3$ receptor antagonists may be ineffective in repeat doses for persistent PONV[9]. Generally, repeat doses of antiemetic agents with similar mechanisms are ineffective, especially when compared to adding a new medication with a unique mechanism of action. Apfel et al. showed that there was an incremental decrease in PONV with the use of ondansetron, dexamethasone, droperidol, and the avoidance of volatile anesthetic agents[8]. It would be reasonable to expect aprepitant to further decrease PONV when used in a multimodal approach for the prophylaxis of PONV, as it is mechanistically different from other antiemetic agents. The use of aprepitant in our series was associated with a very low incidence of vomiting.
